# Landscape of bronchopulmonary dysplasia: from mechanisms to management

**DOI:** 10.3389/fped.2026.1861413

**Published:** 2026-07-22

**Authors:** Ya Pan, Xueli Cheng, Feng Tang, Jiang Lan

**Affiliations:** 1 Department of Neonatology, Shenzhen Longhua Maternity and Child HealthCare Hospital, Shenzhen, Guangdong Province, China; 2 Department of Emergency, People’s Hospital of Longhua, Shenzhen, Guangdong Province, China

**Keywords:** bronchopulmonary dysplasia, chronic lung disease of prematurity, lung, lung disease, lung injury

## Abstract

**Introduction:**

Bronchopulmonary dysplasia (BPD) is the most common chronic lung disease of prematurity and remains a leading cause of morbidity in extremely preterm infants. Despite advances in neonatal intensive care, BPD continues to be associated with substantial respiratory and neurodevelopmental sequelae. This review summarizes current evidence regarding the pathophysiology, risk factors, and therapeutic strategies for BPD.

**Methods:**

A comprehensive search of PubMed, Web of Science, and Embase was conducted using the terms “bronchopulmonary dysplasia”, “premature infants”, “pathophysiology”, “risk factors”, and “treatment”. English-language studies published up to 2025 were screened, and relevant original studies, reviews, and metaanalyses were included for narrative synthesis.

**Results:**

Current evidence indicates that BPD is a multifactorial disorder characterized by impaired alveolarization and pulmonary vascular development resulting from the interaction of prenatal and postnatal injuries. Key pathogenic pathways include oxidative stress, inflammation, dysregulated angiogenesis, and abnormal tissue repair. Prenatal factors such as chorioamnionitis, placental dysfunction, fetal growth restriction, and genetic susceptibility increase vulnerability, while postnatal exposures including oxygen toxicity, mechanical ventilation, infection, and inadequate nutrition further contribute to disease progression. Evidence-based preventive and therapeutic approaches include lung-protective ventilation, early surfactant therapy, caffeine administration, cautious use of postnatal corticosteroids, and optimization of nutritional support. Nevertheless, targeted therapies remain limited.

**Conclusions:**

BPD develops through complex interactions between developmental immaturity and multiple injurious exposures before and after birth. Improved understanding of disease mechanisms and risk stratification may enhance prevention and early intervention, while future research should focus on targeted therapies to improve both short- and long-term outcomes in preterm infants.

## 1 Introduction

**Figure 1:**
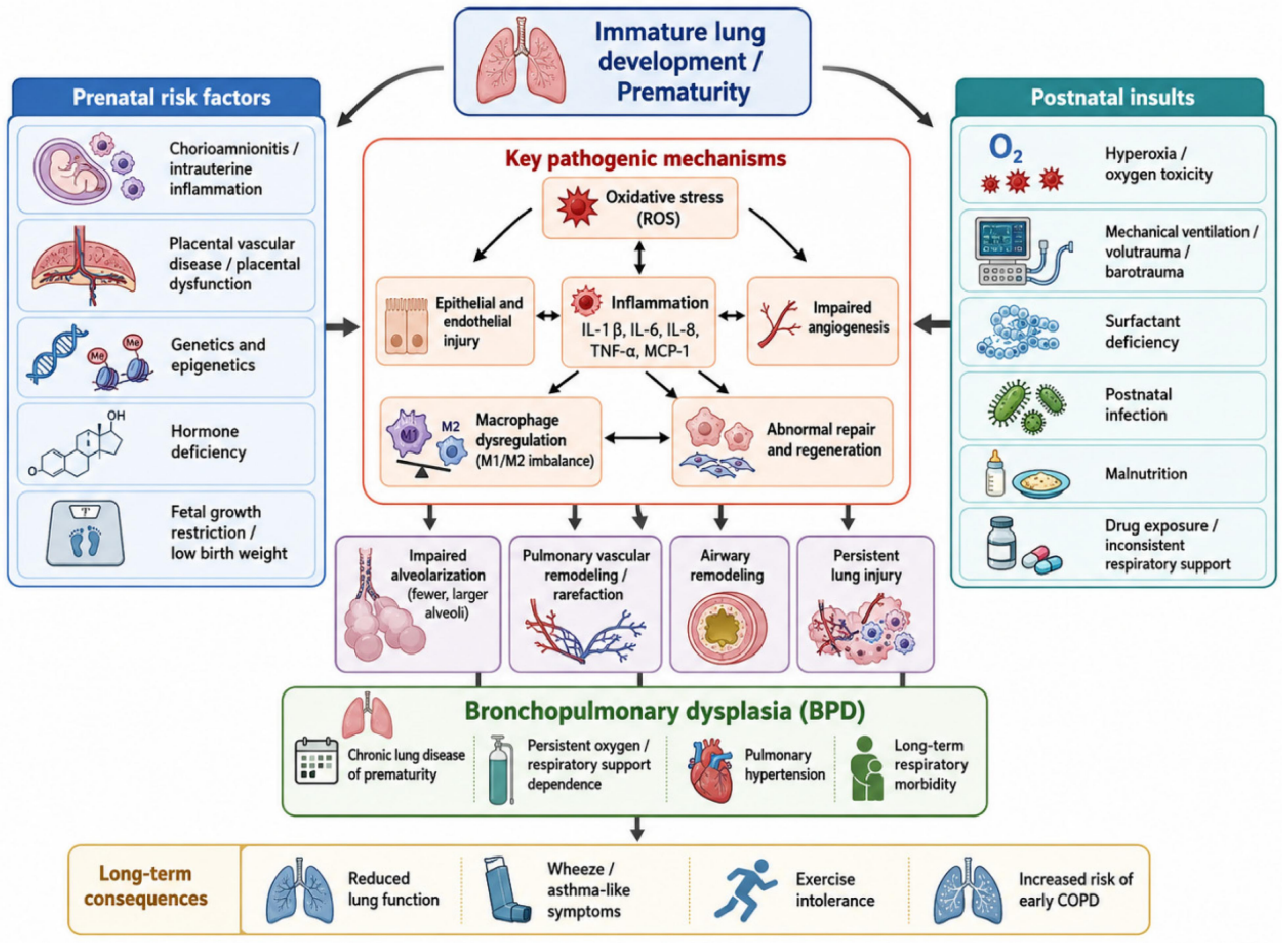
Schematic diagram of bronchopulmonary dysplasia (BPD) pathogenesis.

Bronchopulmonary dysplasia (BPD) is a chronic lung disease of prematurity characterized by persistent respiratory support or oxygen dependence at 36 weeks' postmenstrual age, and it is commonly classified into mild, moderate, and severe forms according to National Institute of Child Health and Human Development (NICHD) 2001 and Jensen 2019 criteria (1). According to the NICHD 2001 and Jensen 2019 criteria, over 50% of infants are diagnosed with BPD, and over 60% of infants have moderate to severe BPD, resulting in a heavy disease burden of BPD in China (2). With improved understanding of BPD pathogenesis and advances in neonatal care, mortality has decreased due to prenatal corticosteroids, advanced respiratory support, and surfactant therapy; however, the incidence of BPD has remained stable or slightly increased (3). Recent studies have confirmed that BPD is a systemic disease with lifelong effects on adult health and quality of life (4). Adults with BPD have abnormal lung function, lower exercise tolerance in tests, and a higher risk of developing chronic obstructive pulmonary disease (5). The clinical characteristics of BPD have undergone significant changes in the past 50 years (6), with profound implications on clinical research (7). BPD represents a spectrum of lung developmental disorders with complex clinical manifestations. This review summarizes current evidence on the epidemiology, pathophysiology, and management of BPD, aiming to improve prevention strategies and clinical outcomes in affected infants. Figure 1 provides a schematic overview of the major pathogenic mechanisms of BPD, integrating prenatal and postnatal risk factors, inflammatory injury, impaired alveolar development, and aberrant repair processes.

## 2 Epidemiological investigation

The incidence of BPD is inversely correlated with gestational age and birth weight, a relationship consistently demonstrated across multiple epidemiological cohorts. Despite advances in neonatal care, BPD remains the most prevalent complication of preterm birth, and its overall prevalence has not declined, largely attributable to the increased survival of extremely preterm infants (2, 8). Notably, the reported incidence of BPD exhibits substantial inter-center variability worldwide, ranging from 20% to 75% (9, 10). This wide disparity is likely multifactorial, reflecting international differences in target population characteristics, perinatal healthcare delivery, application of diagnostic criteria, data quality and integrity, and variations in clinical management protocols.

Preterm birth and low birth weight are the principal risk factors for BPD. In addition, prenatal factors—including inflammation (11), placental vascular disease (12), hormonal deficiencies (13), genetic susceptibility (14), and epigenetic modifications (15)—can disrupt normal lung development, whereas postnatal exposures such as bacterial or viral infections (16), invasive mechanical ventilation (17), oxygen supplementation (18), medications (4, 7), and suboptimal nutrition (19) may exert both acute and long-term adverse effects on pulmonary tissue. As a result, infants with BPD demonstrate considerable heterogeneity in clinical manifestations and long-term outcomes (20). In comparison with full-term infants, preterm infants show normal alveolar volumes but reduced diffusion capacity, indicating impaired alveolarization. Those with BPD have a higher incidence of respiratory illness during infancy and early childhood. Importantly, preterm infants, regardless of BPD status, exhibit poorer lung function than their term counterparts (21). Moreover, children with BPD are at increased risk of asthma-like symptoms during preschool and school ages (22). Respiratory viral infections, particularly respiratory syncytial virus and influenza, often exacerbate underlying pulmonary disease and further elevate the risk of hospitalization in young children (23). Evidence indicates that lung function in preterm infants declines progressively with age, and early-life impairment has been linked to the development of chronic obstructive pulmonary disease (COPD) in adulthood; therefore, the risk of early-onset COPD may also be elevated in children with BPD (24). Clinically, more detailed risk stratification—incorporating gestational age, lung function, genetic background, and environmental factors—is needed to identify preterm infants at greatest risk for respiratory symptoms in childhood and adulthood, thereby enabling earlier targeted intervention.

The NICHD Neonatal Research Network (NRN) criteria, as defined by Jensen et al. in 2019, have been recognized as the most accurate classification system for predicting late death or severe respiratory disease, as well as late death or moderate-to-severe neurodevelopmental impairment (NDI). These criteria were developed and validated in a cohort of 2,677 preterm infants born at <32 weeks' gestation (25). Although the initial validation study demonstrated significantly superior predictive performance over the earlier NICHD 2001 criteria (26) and the commonly used clinical definition, this advantage has not been consistently corroborated across 11 subsequent studies involving diverse patient populations. Although evidence suggests that postponing the assessment to 40 weeks of postmenstrual age (PMA) may improve the prediction of long-term respiratory and neurodevelopmental outcomes (25), this approach is not always practical, as a substantial proportion of infants would have already met discharge criteria by that time. While the physiological definition of BPD reduces clinical bias and facilitates inter-institutional benchmarking, data from preterm birth and respiratory outcome studies indicate that even in prospective cohorts, the performance of O_2_-reduction trials can be inconsistent; therefore, this method is unlikely to gain widespread acceptance in routine practice. Furthermore, using prescription-based criteria to define the disease process is problematic, as such definitions are subject to modification by clinician preferences. Continued reliance on respiratory support at 36 weeks PMA may reflect other pathophysiological processes—such as apnea of prematurity, feeding difficulties, and poor weight gain—rather than intrinsic respiratory dysfunction. When respiratory rate, work of breathing, and weight gain trajectory are the key parameters under evaluation, maintaining saturation within a defined target range (as employed in physiological definitions) serves merely as a surrogate measurement. Importantly, these definitions do not account for the use of medications such as diuretics or corticosteroids, which can influence the degree of O_2_ supplementation required.

Currently, although the models used to define BPD may aid in identifying and stratifying populations at significant risk of disease development, they do not provide specific insights into the pathophysiological processes that drive or perpetuate respiratory failure in individual patients. Recently, Wu and colleagues described three distinct yet overlapping clinical phenotypes of neonatal lung disease in a retrospective study of infants with grade 2–3 BPD (20). The extent of involvement of the lung parenchyma, large airways, and pulmonary vasculature was objectively assessed using chest computed tomography (CT), bronchoscopic findings, sustained dependence on positive airway pressure, and echocardiographic indicators of pulmonary arterial hypertension (PH). Although most infants with BPD were found to have involvement of multiple pulmonary compartments, phenotypes characterized by BPD-associated PH and large airway disease were associated with significantly higher morbidity and mortality. These observations are consistent with earlier reports linking BPD-PH and large airway disease to adverse outcomes. While the NRN definition emphasizes flow dependence rather than supplemental O_2_ requirements—distinguishing grade 2 from grade 1 BPD—and may thereby reflect differential contributions of central airway injury vs. parenchymal disease to outcomes, it does not address pulmonary vascular pathology. Consequently, infants meeting the NRN criteria for grade 2–3 BPD constitute a relatively heterogeneous group, with marked variability in individual prognosis and treatment response. The endpoint of respiratory support at 36 weeks PMA does not capture a single, discrete disease entity, but rather represents the convergence of multiple pathophysiological mechanisms—superimposed upon the normal processes of somatic growth and pulmonary development. Several of the adverse processes that influence the ongoing need for supplemental oxygen and/or airway distending pressure may have originated *in utero* or shortly after birth. Given that there may be a limited window during which experimental therapies are most likely to yield meaningful benefit, early risk stratification and phenotype characterization are essential to identify those infants most likely to respond to novel interventions at the stage of maximum therapeutic efficacy.

## 3 Pathophysiology

### 3.1 Immature lung development

The imbalance between lung injury and repair, driven by inflammatory responses in the immature lung, constitutes a key pathogenic event in the development of BPD. Breathing after birth depends on the structural integrity of the pulmonary saccules and alveoli, with the close interface between alveolar epithelial cells and pulmonary microvascular endothelial cells enabling efficient gas exchange. Lung development commences between the third and sixth weeks of gestation, when endodermal cells give rise to the trachea and lung buds. These buds subsequently invade the surrounding mesenchyme and undergo branching morphogenesis to form the conducting airways and acini, ultimately generating the complex architecture of the mature lung. During the late gestational period, the acini expand and produce alveoli to meet the respiratory demands after birth, as the lung transitions from a solid glandular structure to an open alveolar network capable of gas exchange (27). In the final trimester, the formation of secondary septae increases alveolar number and thereby expands the surface area available for gas exchange. Peripheral lung epithelial cells differentiate into alveolar type 1 (AT1) and alveolar type 2 (AT2) cells, with AT2 cells being responsible for surfactant production. Because preterm infants often initiate ventilation during the canalicular or saccular stages of lung development—before alveolar differentiation is complete—inadequate AT2 cell maturation may lead to surfactant deficiency and respiratory distress syndrome.

After birth, assisted ventilation and oxygen supplementation provide vital respiratory support, and exogenous surfactant administration facilitates the transition to air breathing and improves survival; however, these interventions may also increase the risk of chronic lung disease (28). In summary, postnatal adaptation of the preterm lung to air respiration is frequently compromised by early injury resulting from surfactant deficiency, hyperoxic exposure, mechanical ventilation, malnutrition, infection, and inflammation. This not only impedes normal lung growth and development but may also cause irreversible loss of alveolar surface area, with potentially lifelong consequences.

### 3.2 Acute lung tissue injury

Preterm infants have limited antioxidant capacity and are frequently exposed to hyperoxic environments after birth, leading to the generation of large quantities of reactive oxygen species (ROS) and disruption of the oxidative–antioxidant balance. This cascade of oxidative injury results in DNA strand breaks, lipid peroxidation, excessive apoptosis, and impaired angiogenesis—all of which adversely affect lung development and ultimately contribute to the pathogenesis of BPD (11, 16).

The progression from neonatal lung injury to BPD differs fundamentally from acute lung injury in adults. In preterm infants, the initial insult can cause sustained pulmonary damage, driving persistent proliferation of airway smooth muscle and epithelial cells, which in turn promotes airway remodeling. Moreover, early inflammatory infiltration leads to airway hyperresponsiveness and long-lasting impairment of lung function that may persist into adulthood (29). Although most extremely preterm infants eventually achieve relatively normal lung structure, they remain at increased risk of respiratory and cardiovascular morbidity throughout life. The incomplete understanding of the complex interplay between lung development, injury, and repair has limited the development of effective therapeutic strategies.

### 3.3 Abnormal repair after lung tissue injury

Inflammation represents a common pathogenic pathway converging on the BPD phenotype (30). Initially, the expression of pro-inflammatory mediators is upregulated, neutrophils are recruited into the pulmonary compartment, and alveolar macrophages undergo polarization from the M2 to the M1 phenotype. The resulting M1 macrophages promote the release of pro-inflammatory cytokines, thereby amplifying the inflammatory response and ultimately creating an imbalance between pro-inflammatory and anti-inflammatory mediators. Specifically, levels of interleukin-8 (IL-8), IL-1β, IL-6, tumor necrosis factor-α (TNF-α), monocyte chemoattractant protein-1 (MCP-1), MCP-2, and MCP-3 are elevated, whereas macrophage inflammatory protein-1α (MIP-1α), MIP-1β, and IL-10 are decreased, collectively contributing to inflammatory tissue injury.

Most extremely preterm infants with chorioamnionitis are often exposed to antenatal corticosteroids, which, as demonstrated in animal models, suppress chorioamnionitis-associated inflammation but may also impair alveolar formation (31). Premature infants who are exposed to a variety of prenatal and postnatal inflammatory modulators may exhibit altered injury and repair pathways, thereby accounting for the marked clinical variability and severity of BPD observed across individuals (16, 30).

The pathophysiological cascade of BPD is summarized in Figure 1, illustrating the interaction between inflammation, oxidative stress, and disrupted lung development and repair.

Schematic overview of BPD pathogenesis. Prenatal factors (chorioamnionitis, placental dysfunction, genetic susceptibility) and postnatal insults (oxygen toxicity, mechanical ventilation, infection, and malnutrition) trigger oxidative stress and inflammatory responses. These lead to epithelial and endothelial injury, impaired alveolarization, vascular remodeling, and dysregulated lung repair, ultimately resulting in chronic lung disease of prematurity.

## 4 Prevention and treatment methods

### 4.1 Short-term management

#### 4.1.1 Avoid tracheal intubation and mechanical ventilation

To minimize lung injury in extremely preterm infants, it is advisable to avoid endotracheal intubation and invasive mechanical ventilation in the immediate postnatal period. Instead, non-invasive respiratory support modalities—including nasal continuous positive airway pressure (NCPAP), non-invasive positive pressure ventilation (NIPPV), and high-flow nasal cannula (HFNC)—should be considered, all of which appear to be similarly effective in reducing the incidence of BPD. Randomized controlled trial (RCT) and meta-analysis data support early implementation of CPAP therapy in infants at risk of BPD in the delivery room (32). A follow-up study demonstrated a significant association between the use of CPAP and a reduced incidence of respiratory morbidity in preterm infants (33). When invasive mechanical ventilation cannot be avoided, volume-targeted ventilation (VTV) should be strongly considered. Overinflation, atelectasis, and oxygen toxicity are key contributors to ventilator-induced lung injury (VILI). Accordingly, lung-protective ventilation strategies should aim to optimize lung volume (to prevent atelectasis), limit tidal volume, and minimize oxygen exposure.

VTV and high-frequency ventilation most closely align with the principles of lung-protective ventilation; both modalities stabilize tidal volume delivery, reduce lung injury, and lower the incidence of BPD in preterm infants with respiratory distress syndrome (34). A further retrospective study demonstrated a strong association between the duration of invasive mechanical ventilation and the composite outcome of BPD or death (35). Overall, current evidence continues to support early weaning from invasive mechanical ventilation within the first week of life, and extubation trials should be considered in infants who tolerate weaning to low ventilator settings, even though long-term success rates may be modest (36).

#### 4.1.2 Early respiratory support and surfactant administration

Delivery room interventions aimed at preventing BPD have been shown to influence long-term outcomes (37). Early recovery strategies are associated with improved outcomes in full-term infants; however, their benefit remains uncertain in preterm populations (38). No correlation has been observed between the use of higher oxygen saturation targets (>90%) and an increased incidence of “physiological” BPD (39). A population-based outcome study of extremely preterm infants in Germany from 2010 to 2017 found that targeting a lower saturation range (85%–89%) was associated with an elevated risk of pre-discharge mortality (40). More recently, a meta-analysis of five high-quality randomized controlled trials comparing high (91%–95%) and low (85%–89%) saturation targets has provided clinically useful guidance. The higher target range was associated with reduced risks of death and necrotizing enterocolitis, whereas no difference was observed between groups in the composite outcome of death or severe disability at 18–24 months of corrected age. Treatment for retinopathy of prematurity was more frequent in the high-saturation group. Importantly, there was no significant difference in the incidence of BPD between the two groups. The currently recommended target oxygen saturation range for neonatal intensive care units in the United States is 90%–95% (41).

Exogenous surfactant therapy has been shown to improve neonatal outcomes. Historically, surfactant was administered via endotracheal intubation; however, in recent years, several alternative techniques have been developed to deliver surfactant to infants receiving non-invasive respiratory support, including minimally invasive surfactant administration—often referred to as minimally invasive surfactant therapy (MIST) (42). A recent study demonstrated that compared with traditional intubation-based surfactant delivery, administration via a fine catheter was associated with a reduced composite outcome of death or BPD. Nevertheless, the quality of published systematic reviews has generally been suboptimal, and these alternative methods require further rigorous evaluation before clinical implementation. Definitive evidence from large-scale randomized controlled trials is needed to determine whether this approach should be adopted for routine use in neonatal intensive care (43).

#### 4.1.3 Caffeine

The use of respiratory stimulants, such as caffeine, represents one of the most effective pharmacological strategies for reducing the risk of BPD. Early initiation of caffeine treatment within the first 3 days after birth has been shown to significantly reduce the incidence of both BPD and apnea (44). A retrospective controlled study demonstrated that in infants born after 28 weeks of gestation, early caffeine administration (within 24 h of birth) was associated with a 1.34-day reduction in the duration of invasive mechanical ventilation and a lower incidence of moderate-to-severe BPD (45). In addition, early caffeine exposure has been found to attenuate hyperoxia-induced hippocampal injury in neonatal rats, suggesting potential neuroprotective properties (46). Caffeine also reduces the requirement for mechanical ventilation by improving lung function, enhancing diaphragmatic activity, and increasing central chemosensitivity to carbon dioxide. A meta-analysis of 13 randomized controlled trials evaluating different maintenance doses of caffeine reported that the high-dose group (10–20 mg/kg/day) had higher rates of successful extubation and treatment effectiveness, as well as lower extubation failure rates, compared with the lower-dose group (47). Furthermore, the high-dose caffeine group exhibited a significantly reduced incidence of moderate-to-severe BPD and a more pronounced increase in heart rate (48).

#### 4.1.4 Postpartum steroids

Prenatal corticosteroids accelerate fetal lung maturation and reduce the risks of perinatal mortality, neonatal mortality, and respiratory distress syndrome. However, the role of postnatal corticosteroids in preventing and alleviating BPD remains contentious. A meta-analysis of 26 randomized controlled trials involving 4,167 infants demonstrated that early postnatal corticosteroid administration (<8 days) was associated with shorter durations of mechanical ventilation and a reduced incidence of BPD, but also with an increased risk of adverse neurological sequelae (49). Early (≤7 days) treatment with moderate-to-high-dose dexamethasone in ventilator-dependent preterm infants reduces pulmonary inflammation and facilitates extubation, thereby lowering BPD risk; however, it is also linked to short-term adverse effects, including intestinal perforation and growth impairment. Dexamethasone exerts purely glucocorticoid activity, whereas hydrocortisone activates both glucocorticoid and mineralocorticoid receptors, albeit with weaker potency. Studies have shown that early low-dose hydrocortisone improves neonatal mortality and BPD-free survival in infants exposed to chorioamnionitis (50). Compared with dexamethasone, hydrocortisone has weaker glucocorticoid efficacy but possesses mineralocorticoid activity, which may theoretically confer better neurological safety. Nevertheless, its effectiveness and safety in the broader preterm population await confirmation through high-quality randomized controlled trials. A study evaluating low-dose dexamethasone (0.89 mg/kg, DART regimen over 10 days) in infants who remained on mechanical ventilation beyond the first week of life found an increased likelihood of extubation at the end of treatment, but no reduction in BPD. Follow-up assessments revealed neither adverse consequences nor neurodevelopmental benefits, suggesting that a simple low-dose strategy may be insufficient to address the underlying pathophysiology (51). A retrospective study across seven level III neonatal intensive care units reported that the use of more than one course of the DART regimen for extubating ventilator-dependent preterm infants after the first week of life was associated with a higher incidence of cerebral palsy at 2 years of corrected age (52). Budesonide, particularly when administered as surfactant-based intratracheal instillation, appears to be a safe and effective option for BPD prevention, reducing both the incidence of BPD and all-cause mortality at 36 weeks of corrected gestational age (53). However, its long-term neurodevelopmental impact requires further follow-up data for confirmation.

In summary, postnatal corticosteroids should not be considered a universal treatment. Their use must be guided by strict risk stratification and reserved for ventilator-dependent preterm infants at high risk of BPD who are difficult to wean. Clinical decision-making should adhere to the principle of “minimum effective dose and shortest effective course,” and parents should be fully informed of the potential short- and long-term risks.

#### 4.1.5 Diuretic therapy

Although diuretics such as furosemide may theoretically improve respiratory mechanics in infants with BPD by reducing pulmonary edema, their efficacy and safety for the prevention or treatment of BPD remain highly controversial, and clinical evidence is limited. An animal study demonstrated that young rats exposed to hyperoxia developed pulmonary edema, uneven alveolar distribution, and septal thinning (54). In a large multicenter retrospective cohort study, a longer duration of furosemide exposure was significantly correlated with a lower incidence of BPD. However, given the observational design, a causal relationship cannot be established, and this association may be substantially confounded—for instance, sicker infants may be more likely to receive diuretic therapy (55). Diuretic use is associated with well-documented short- and long-term risks, including electrolyte disturbances (e.g., hyponatremia and hypokalemia), renal calcification, ototoxicity (particularly when combined with certain antibiotics), and potential exacerbation of metabolic bone disease. These hazards have raised concerns about the overall risk-benefit profile (56). Systematic reviews and meta-analyses have not yielded robust evidence to support the routine use of diuretics for BPD prevention or for improving long-term respiratory outcomes. Currently, diuretics should not be considered a routine preventive or therapeutic strategy for BPD. Their use should be strictly reserved for infants with overt clinical signs of fluid overload, and regarded as a short-term palliative measure rather than a disease-modifying intervention targeting the underlying pathophysiology (57). Overall, the lack of high-quality randomized controlled trials and the absence of supportive evidence argue against routine diuretic therapy. Clinical decisions should be highly individualized, carefully weighing the limited and uncertain benefits against the well-recognized risks.

#### 4.1.6 Nutritional support

The capacity for postnatal lung growth and repair is contingent upon adequate nutritional intake after birth. In extremely low birth weight infants, insufficient protein and energy intake during the first week of life has been associated with poor weight gain by 36 weeks of postmenstrual age and significant deterioration in lung function parameters (58). Ideally, infants with BPD should receive fluid intake not exceeding 135–150 mL/kg/day and energy intake of 120–150 kcal/kg/day (59).

A retrospective cohort study demonstrated that preterm infants who developed BPD had significantly lower energy and lipid intake, alongside higher fluid intake, during the early postnatal period. Early nutrition and energy intake within the first week of life were significantly correlated with the subsequent development of BPD (60). Therefore, strong consideration should be given to relative fluid restriction, early initiation of enteral feeding, and optimization of parenteral nutrition composition. A randomized controlled trial showed that exclusive breastfeeding was significantly associated with a reduced risk of BPD compared with formula supplementation (61). In the management of infants with evolving or established BPD, exclusive use of breast milk—preferably fresh—is recommended.

### 4.2 Long-term management

Although early respiratory care strategies have advanced substantially in minimizing ventilator-associated lung injury from birth through the later stages of neonatal intensive care, some preterm infants continue to experience severe chronic respiratory disease and associated complications both during their neonatal intensive care unit (NICU) stay and after discharge. Consequently, there remains an ongoing need for continued improvement in long-term management approaches.

Long-term care needs encompass tracheostomy, prolonged mechanical ventilation, high inspired oxygen fractions, and a range of respiratory medications, including intermittent corticosteroids, bronchodilators, and diuretics (62). According to National Institutes of Health (NIH) criteria, preterm infants requiring ongoing respiratory support at 36 weeks of corrected age—including those with tracheostomy and chronic ventilator dependence—represent the most severe subgroup of BPD. These infants are at the highest risk of late mortality, cardiovascular dysfunction, and extensive comorbidities, as well as poor somatic growth, neurocognitive impairment, and developmental delay (63). Better characterization of the key pathophysiological drivers in individuals with severe BPD may enhance overall management and enable more selective, targeted pharmacotherapy.

In the context of severe BPD, the goals and challenges of managing cardiopulmonary dysfunction differ substantially from those focused primarily on prevention and short-term treatment (63, 64). Despite some areas of consensus, considerable variation exists across centers in respiratory care, diagnostic evaluation, medication selection and use, and long-term follow-up practices, reflecting differences in institutional approaches and patient populations (64). In the absence of multicenter randomized controlled trial data, current long-term management strategies for severe BPD remain largely based on clinical experience, physiological principles, and small case series. As a result, the optimal approach to improving long-term outcomes in this high-risk population remains uncertain.

Beyond the lack of proven, evidence-based strategies to improve long-term outcomes, the management of chronic lung disease differs fundamentally from that of acute respiratory failure, particularly with respect to mechanical ventilation approaches (65). In the setting of complex chronic lung disease, communication barriers among providers, specialists, caregivers, and family members during prolonged hospitalization may lead to inconsistent care and adverse outcomes. Interdisciplinary care teams have the potential to mitigate many of these challenges and improve infant outcomes. Establishing such teams may help enhance survival, shorten hospital stays, and reduce healthcare costs (66). The clinical course of infants with severe BPD is complex and associated with multiple comorbidities. These infants are frequently rehospitalized throughout childhood and often experience poor continuity of care. Therefore, bridging inpatient and long-term outpatient care is essential to improve care coordination, understand the complex clinical trajectory, and anticipate complications arising from persistent disease. Although the developing lung possesses considerable remodeling capacity, abnormalities of the airways, airspaces, and vasculature may persist. Multiple studies have demonstrated that lung function and structural deficits continue through childhood, adolescence, and young adulthood in patients with BPD. Furthermore, some studies have emphasized that preterm young adults—regardless of BPD history—are at risk for cardiac dysfunction (67). Multiple reports have highlighted that preterm infants are at high risk of developing severe respiratory dysfunction during childhood (21). These reports also underscore the importance of early intervention, advocating for integration of early NICU care with close outpatient follow-up and enhanced collaboration between pediatric and adult subspecialists, particularly in pulmonary and cardiovascular medicine. Clearly, further research is needed to define optimal strategies for improving lung and cardiovascular health across the lifespan of preterm infants with and without severe BPD, ultimately enhancing their quality of life.

Advances in imaging, transcriptomics, epigenetics, and lineage-tracing technologies have provided deeper insights into the molecular processes governing diverse cell types—including stem and progenitor cells—and their interactions in lung formation and regeneration (68, 69). These approaches have helped identify signaling pathways that guide lung development, repair, and function, as well as transcriptional networks that may enable the discovery of relevant biomarkers, and have informed clinical strategies aimed at protecting key progenitor cells and the processes essential for lung formation and repair (70, 71). Such insights will facilitate the development of therapeutic approaches that promote normal tissue regeneration while inhibiting pathological remodeling and the loss of cells and matrix components—processes that underlie many chronic lung diseases. Despite severe early lung dysfunction and prematurity-related injury, most infants with BPD show significant recovery of lung structure and function over time. A better understanding of the resilience of the developing lung will provide further insights into both normal and pathological repair processes relevant to chronic lung diseases across the entire lifespan.

Although malnutrition is recognized as a contributing factor in BPD pathogenesis, nutritional interventions studied to date—including high amino acid or early lipid supplementation in parenteral nutrition, inositol, N-acetylcysteine, and iodine—have not been shown to be effective (72). Accordingly, well-designed randomized controlled trials of nutritional strategies are needed to evaluate BPD-related outcomes and mortality.

Although inflammation resulting from chorioamnionitis and neonatal infections may exacerbate lung injury, antibiotic administration before or after delivery in uninfected preterm infants has not been shown to reduce BPD risk (73, 74). Given the biological plausibility of these interventions, further exploration is required to refine treatment approaches. We are unable to evaluate interventions that have not been examined within the context of systematic reviews, as many are either in early-phase human trials or have not been studied for their potential role in BPD prevention. An emerging intervention—stem cell therapy—holds promise. A recent meta-analysis of 25 preclinical studies demonstrated that mesenchymal stem cell or conditioned medium therapy significantly improved alveolarization and reduced inflammation and fibrosis in rodent models of hyperoxia-induced BPD. In addition, multiple ongoing trials are evaluating stem cell-based therapies for BPD (75). However, several aspects—including the optimal cell source, dosage, route of administration, and timing of treatment—remain to be determined. Moreover, long-term safety must be confirmed before stem cell therapy can be translated into routine clinical practice. Table 1 summarizes current prevention and treatment strategies for BPD, together with their supporting evidence level and key clinical outcomes.

**Table 1:** Prevention and treatment strategies for BPD.

Intervention	Strategy	Evidence level	Key effect on BPD
CPAP/non-invasive ventilation	Avoid invasive ventilation	High (RCT/meta-analysis)	↓ BPD incidence
Surfactant therapy (MIST/LISA)	Early respiratory support	Moderate	↓ Death/BPD composite
Caffeine	Respiratory stimulant	High	↓ BPD, ↓ extubation failure
Postnatal steroids	Anti-inflammatory therapy	Moderate	↓ BPD but neurodevelopment risk
Diuretics	Fluid management	Low	No routine benefit
Nutritional support	Breast milk, energy optimization	Moderate	↓ BPD risk
Stem cell therapy	Experimental regeneration	Preclinical/early trials	Potential benefit

## 5 Conclusions

BPD is a complex, multifactorial lung disease that extends beyond the pulmonary system, encompassing other comorbidities commonly seen in preterm infants, such as neurodevelopmental impairment and growth failure. The absence of a standardized, objective definition for BPD remains a major obstacle to the evaluation of novel therapeutic interventions. As efforts to develop more objective biomarker-based or “omics”-driven diagnostic criteria continue, continued emphasis should be placed on the consistent application of clinical definitions that effectively identify infants at the highest risk of mortality and morbidity. Despite substantial advances in our understanding of BPD pathogenesis, evidence-based therapeutic options remain limited. Consequently, the development of novel strategies aimed at preventing BPD or attenuating its severity holds great promise for improving long-term health outcomes in the growing population of preterm survivors.

## Data Availability

The original contributions presented in the study are included in the article/Supplementary Material, further inquiries can be directed to the corresponding author.
